# Supplementary material for: Multi-hit STAG2 mutations define a high-risk subset of MDS and reveal convergent evolutionary targeting of cohesin
Source: bioRxiv. 2026 Sep 3:2026.09.02.749005. Preprint. [Version 1] doi: 10.64898/2026.09.02.749005 (PMC13684691; doi:10.64898/2026.09.02.749005)
Supplement: Supplementary file 1 [file media-1.pdf]

Supplementary Material

eTable 1. List of genes covered by Cleveland Clinic hematologic neoplasm next-generation sequencing (NGS) panel.

ABL1	ASXL1	BCOR	BCORL1	BRAF	CALR	CBL	CDKN2A
CEBPA	CSF3R	CUX1	DDX41	DNMT3A	EED	ETNK1	ETV6
EZH2	FBXW7	FLT3	GATA1	GATA2	GNAS	IDH1	IDH2
IKZF1	JAK2	JAK3	KDM6A	KIT	KMT2A	KRAS	LUC7L2
MPL	MYD88	NF1	NOTCH1	NPM1	NRAS	PAX5	PHF6
PIGA	PPM1D	PRPF8	PTEN	PTPN11	RAD21	RIT1	RUNX1
SETBP1	SF3B1	SH2B3	SMC1A	SMC3	SRSF2	STAG2	STAT3
STAT5B	SUZ12	TET2	TP53	U2AF1	WT1	ZRSR2	

eTable 2. Comparison of baseline characteristics in multiSTAG2^{MT} and monoSTAG2^{MT} MN cases.

**Variables**	**monoSTAG2^{MT} (n = 195)**	**multiSTAG2^{MT} (n = 38)**	**p-value**
**Age (y)**	73.7 (65.5, 80.7)	71.3 (65.6, 78.8)	0.67
≥60	159 (81.5)	34 (89.5)	0.35
<60	36 (18.5)	4 (10.5)	
**Gender**			0.07
Male	124 (63.6)	30 (79.0)	
Female	71 (36.4)	8 (21.0)	
**WHO Classification (2016)**			
**MDS**	78 (40.0)	22 (57.9)	0.30
MDS-EB-1	25	5	
MDS-EB-2	14	10	
MDS-MLD-RS	4	3	
MDS-U	2	1	
MDS-MLD	28	3	
**MDS/MPN**	12 (6.2)	3 (7.9)	
CMML	9	1	
MDS/MPN-U	1	2	
**AML**	71 (36.4)	11 (29.0)	
sAML	35	8	
tAML	0	1	
pAML	34	2	
**MPN**	7 (3.6)	0 (0.0)	
MF	2	0	
ET/MF	1	0	
PV	1	0	
**Other**	27 (13.9)	2 (5.3)	
CH	5	0	
CCUS	20	2	
**Cytogenetics**			0.50
Normal karyotype	100 (62.1)	19 (55.9)	
Abnormal karyotype	61 (37.9)	15 (44.1)	

eTable 3. Comparison of clinical characteristics in multiSTAG2^{MT} and monoSTAG2^{MT} MN cases.

Variables	monoSTAG2^{MT} (n = 195)	multiSTAG2^{MT} (n = 38)	p-value
**Hematologic parameters**			
WBC (10^9/L) (median/range)	2.0 (1.0, 5.0)	1.0 (1.0, 4.0)	0.42
<4.5 x 10^9/L - leukopenia	112 (60.22)	24 (66.7)	0.47
>11.0 x 10^9/L - leukocytosis	38 (20.4)	5 (13.9)	0.36
Hemoglobin (g/dL)	8.6 (7.6, 9.7)	8.0 (7.5, 9.5)	0.27
<12 g/dL (F) or <13g/dL (M) - anemia	179 (94.2)	35 (97.2)	0.70
>17 g/dL - erythrocytosis	1 (0.54)	0 (0.0)	1.00
Platelet (10^9/L)	68 (36, 139)	64 (29, 120)	0.48
<150 X 10^9/L - thrombocytopenia	144 (77.4)	30 (83.3)	0.43
>450 X 10^9/L - thrombocytosis	9 (4.8)	2 (5.6)	0.69
ANC (10^9/L)	1.39 (0.43, 4.28)	0.85 (0.34, 2.67)	0.12
<1.8 x 10^9/L - neutropenia	115 (61.8)	20 (64.5)	0.87
>6.0 x 10^9/L - neutrophilia	30 (16.1)	2 (5.6)	0.26
**Cytopenias**			
Monocytopenia	25 (13.6)	4 (11.1)	0.66
Bicytopenia	92 (50.0)	11 (30.6)	
Pancytopenia	67 (36.4)	21 (58.3)	
**Bone Marrow Morphology**			
Blasts	3 (3, 30)	9 (3, 18)	0.52
>= 5%	104 (53.3)	23 (60.5)	0.42
Cellularity			0.45
Hypercellular	91 (58.0)	19 (55.9)	
Normocellular	37 (23.6)	11 (32.4)	
Hypocellular	29 (18.5)	4 (11.8)	
M/E ratio	1.7 (1.1, 3.8)	2.6 (1.2, 4.8)	0.28
Myelofibrosis	29 (18.4)	3 (9.7)	0.30
**Dysplastic Lineage**			
Myeloid	74 (45.4)	21 (65.6)	**0.04**
Erythroid	96 (58.9)	24 (72.7)	0.14
Megakaryocytic	112 (70.4)	29 (90.6)	**0.02**
**Number of dysplastic BM lineage**			
None	30 (18.3)	0 (0.0)	**0.02**
Unilineage	28 (17.1)	4 (12.5)	
Bilineage	64 (39.0)	14 (43.8)	
Trilineage	42 (25.6)	14 (43.8)	

eTable 4. Variant allele frequencies of each *STAG2* mutation within the multi-hit cohort.

UPN	VAF 1	VAF 2	VAF 3	VAF 4
1	12.6	4.0		
2	18.7	19.1		
3	35.9	9.4		
4	11.2	6.1	63.2	13.6
5	5.4	12.9		
6	20.9	6.7		
7	4.2	38.3		
8	5.3	12.9	4.2	
9	4.4	6.7		
10	7.8	61.0		
11	6.7	16.8		
12	92.1	5.4		
13	27.4	7.5		
14	32.6	6.4	6.5	6.8
15	5.2	5.8		
16	13.2	81.3		
17	12.1	45.1		
18	10.7	66.2		
19	71.2	13.0		
20	21.4	15.5		
21	17.9	61.5		
22	9.0	63.0	14.0	
23	6.0	6.0	9.0	
24	11.0	13.0	13.0	
25	40.4	47.5		
26	35.9	9.4		
27	4.3	45.1		
28	35.1	5.8		
29	13.6	25.5		
30	7.6	7.8		
31	5.6	69.0		
32	4.8	38.6	12.8	
33	31.2	59.1		
34	7.2	59.2		
35	54.7	17.9		
36	4.3	68.0		
37	34.9	10.2		
38	9.3	6.0		

UPN – unique patient number, VAF – variant allele frequency.

Supplementary Figure Legends

eFigure 1. Clonal hierarchy of the first STAG2 mutation was not associated with reproducible differences in co-mutational patterns, supporting recurrent STAG2 acquisition across diverse genetic backgrounds. Forest plot showing the odds ratios (OR) of genes commonly mutated in myeloid neoplasms among multiSTAG2^{MT} cases for **A)** DOM vs. SEC. **B)** COD vs SEC. **C)** DOM vs. COD clonal rankings.

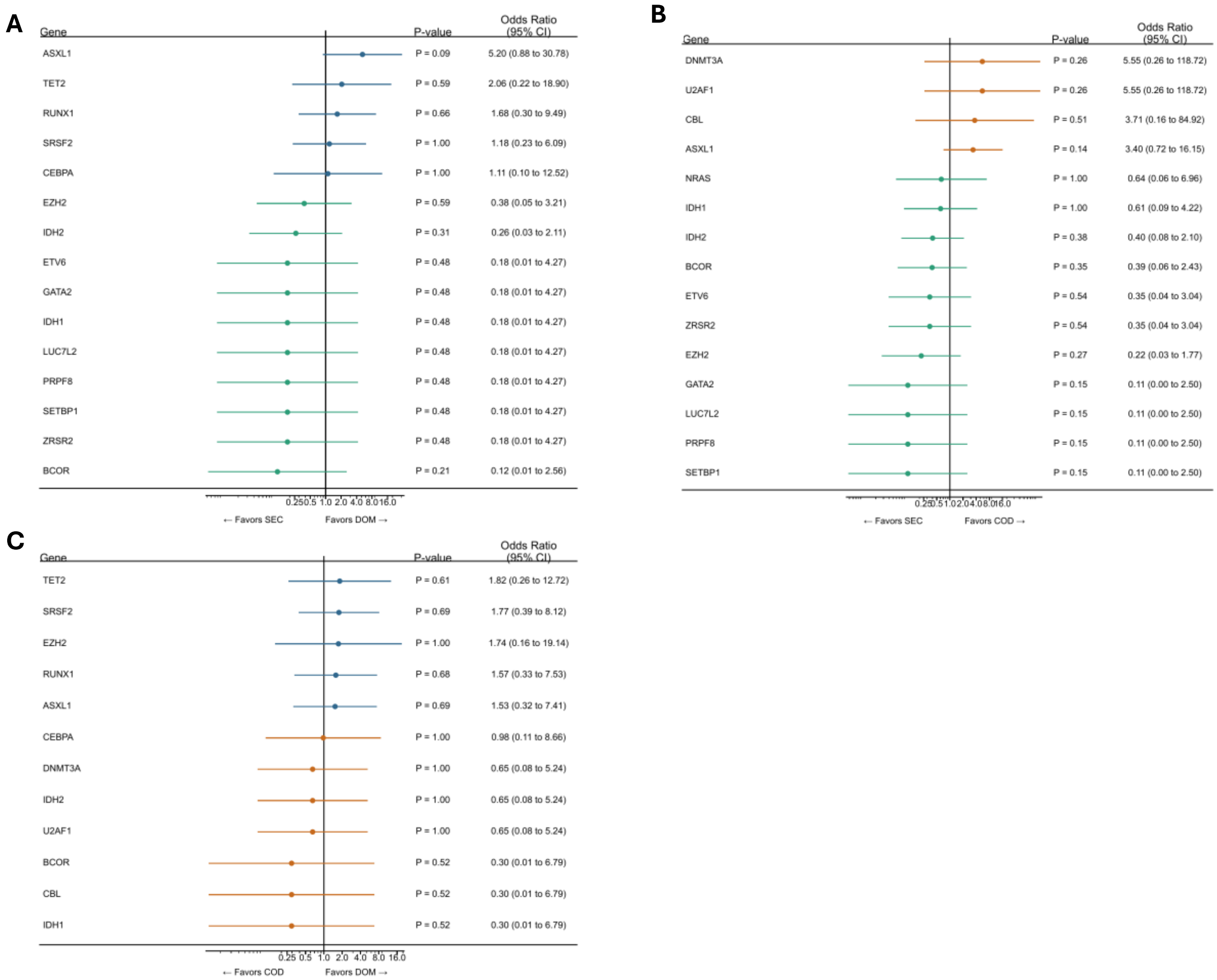

eFigure 2. Among MDS patients with multi-hit STAG2 mutations, dominant first-hit STAG2 clones were associated with higher leukocyte counts, whereas codominant clones were enriched for leukopenia and neutropenia. Box plots displaying the levels of **A)** WBC, **B)** Hemoglobin, **C**) Platelet, **D)** ANC, **E)** BM blast %, **F)** BM cellularity, **G)** Monocytes, and **H)** M/E ratio in multiSTAG2^{MT} cases with MDS based on the clonal status of the 1st STAG2 hit.

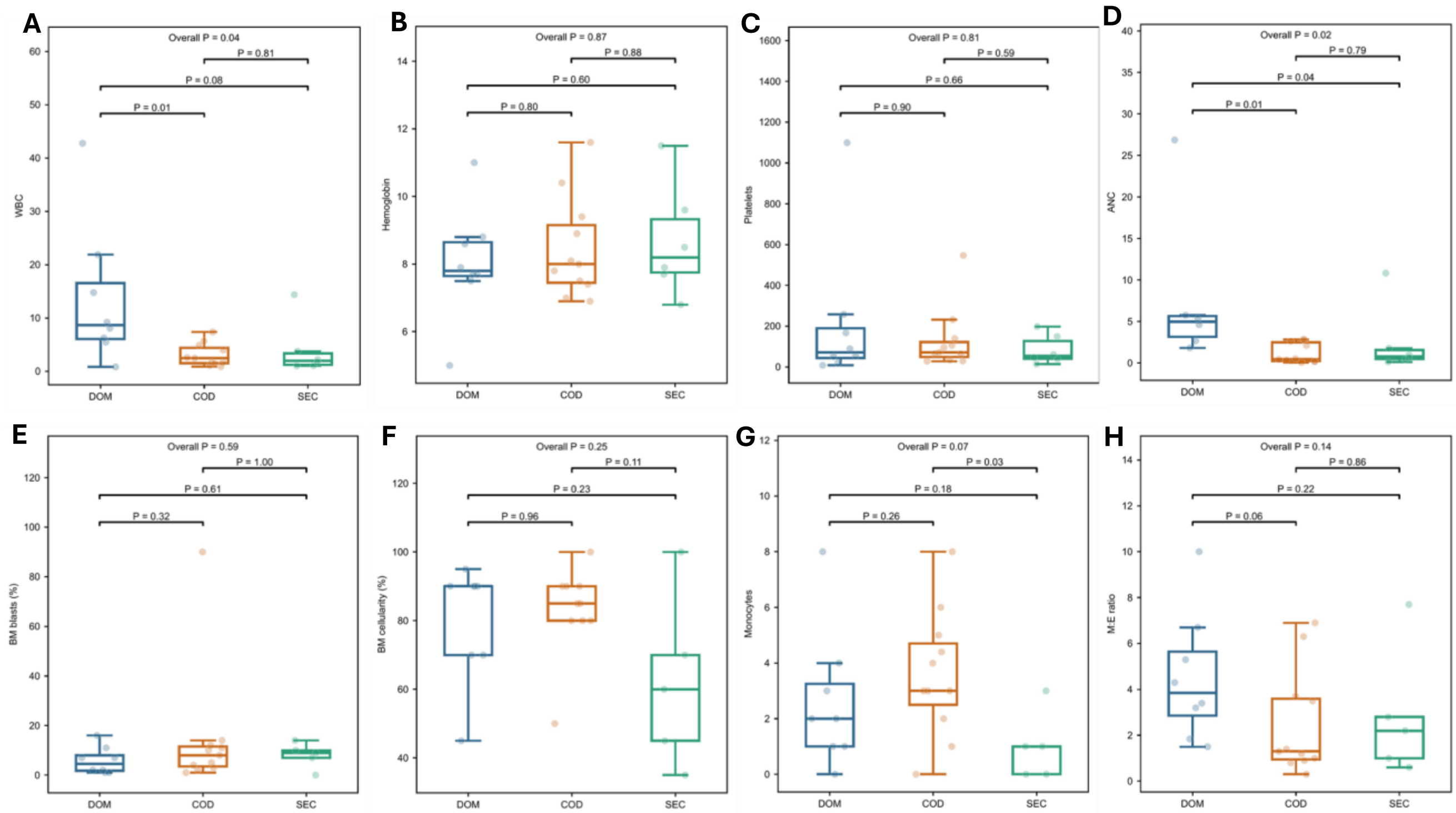

eFigure 3. Clinical and hematologic characteristics were largely similar across clonal hierarchy groups in AML, indicating limited phenotypic impact of STAG2 clonal timing in this disease context. Box plots displaying the levels of **A)** WBC, **B)** Hemoglobin, **C**) Platelet, **D)** ANC, **E)** BM blast %, **F)** BM cellularity, **G)** Monocytes, and **H)** M/E ratio of multiSTAG2^{MT} cases with AML based on the clonal status of the 1st STAG2 hit.

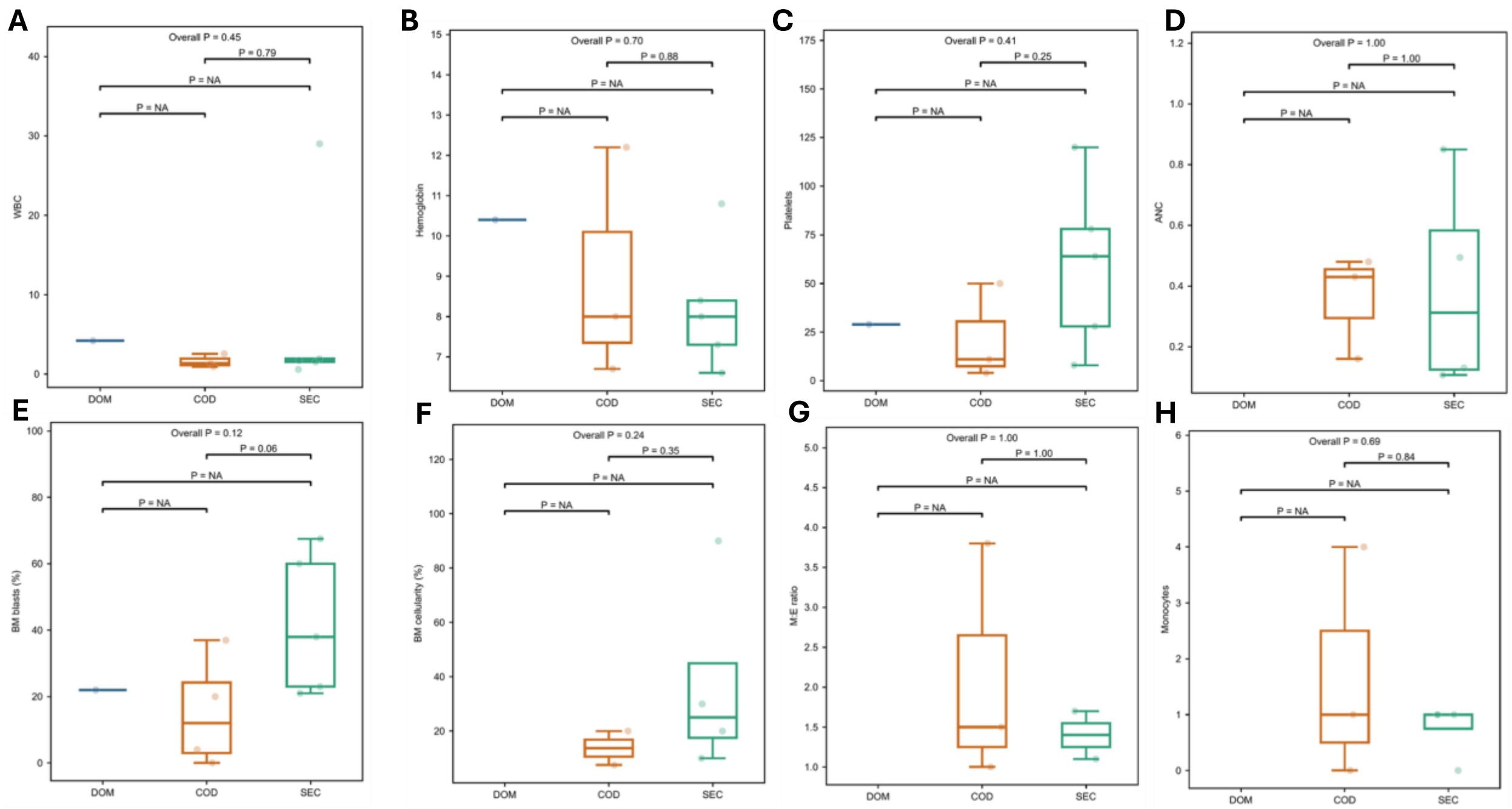

eFigure 4. Independent review of clinical short-read sequencing data confirmed the presence of multiple STAG2 mutations selected for long-read allelic phasing analyses. Patient samples aligned to the reference genome (GRCH38 – hg 38**). A)** UPN 32, and **B)** UPN 33.

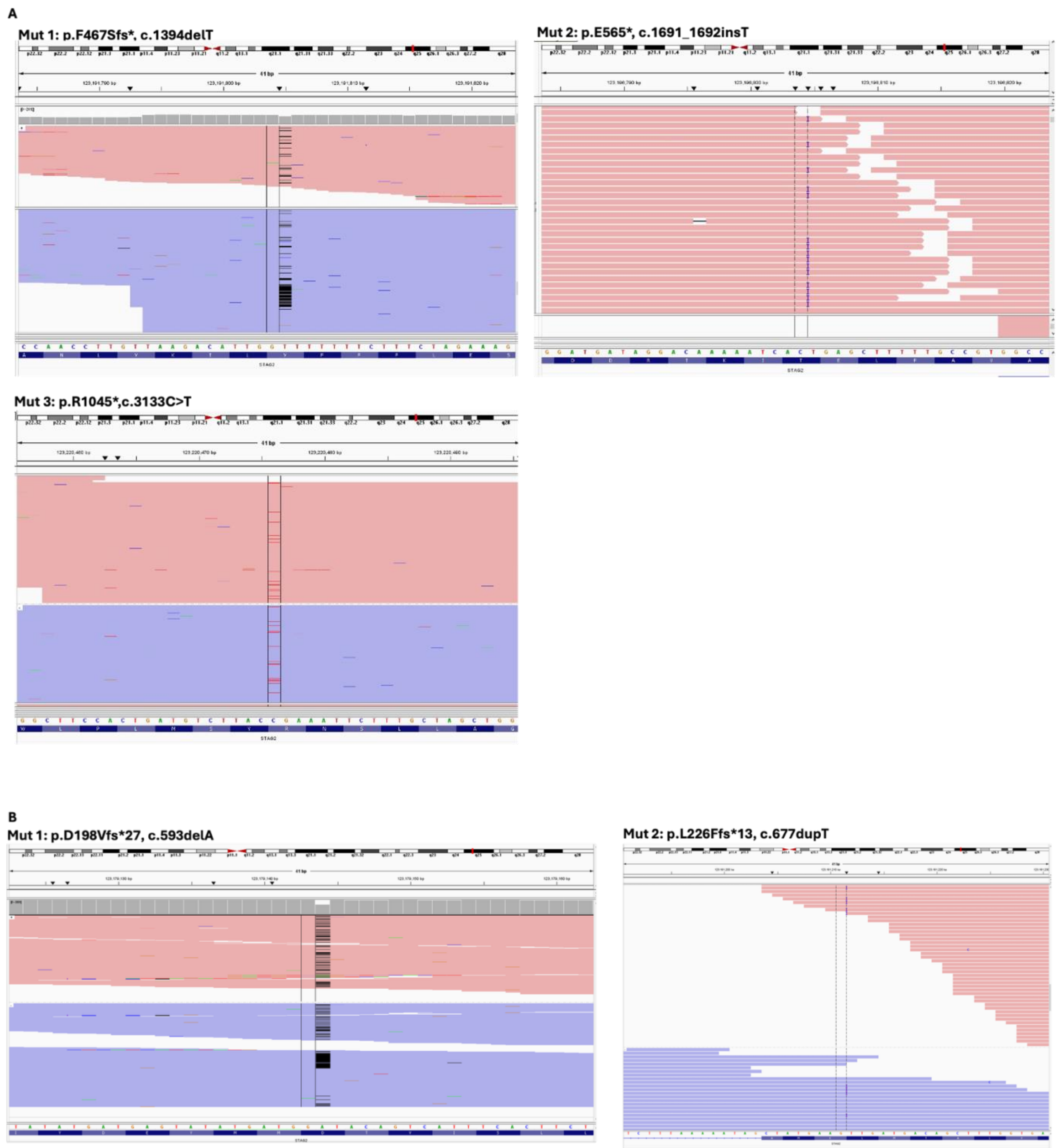

eFigure 5. The adverse prognostic impact of multi-hit STAG2 mutations was restricted to MDS and was not observed in AML, regardless of clonal hierarchy. Kaplan-Meier curves comparing overall survival in **A)** all MN cases, **B)** multi-hit $STAG2^{MT}$ MN cases only, **C)** all MDS cases, **D)** multi-hit $STAG2^{MT}$ MDS cases only, **E)** all AML cases, **F)** multi-hit $STAG2^{MT}$ AML cases only.

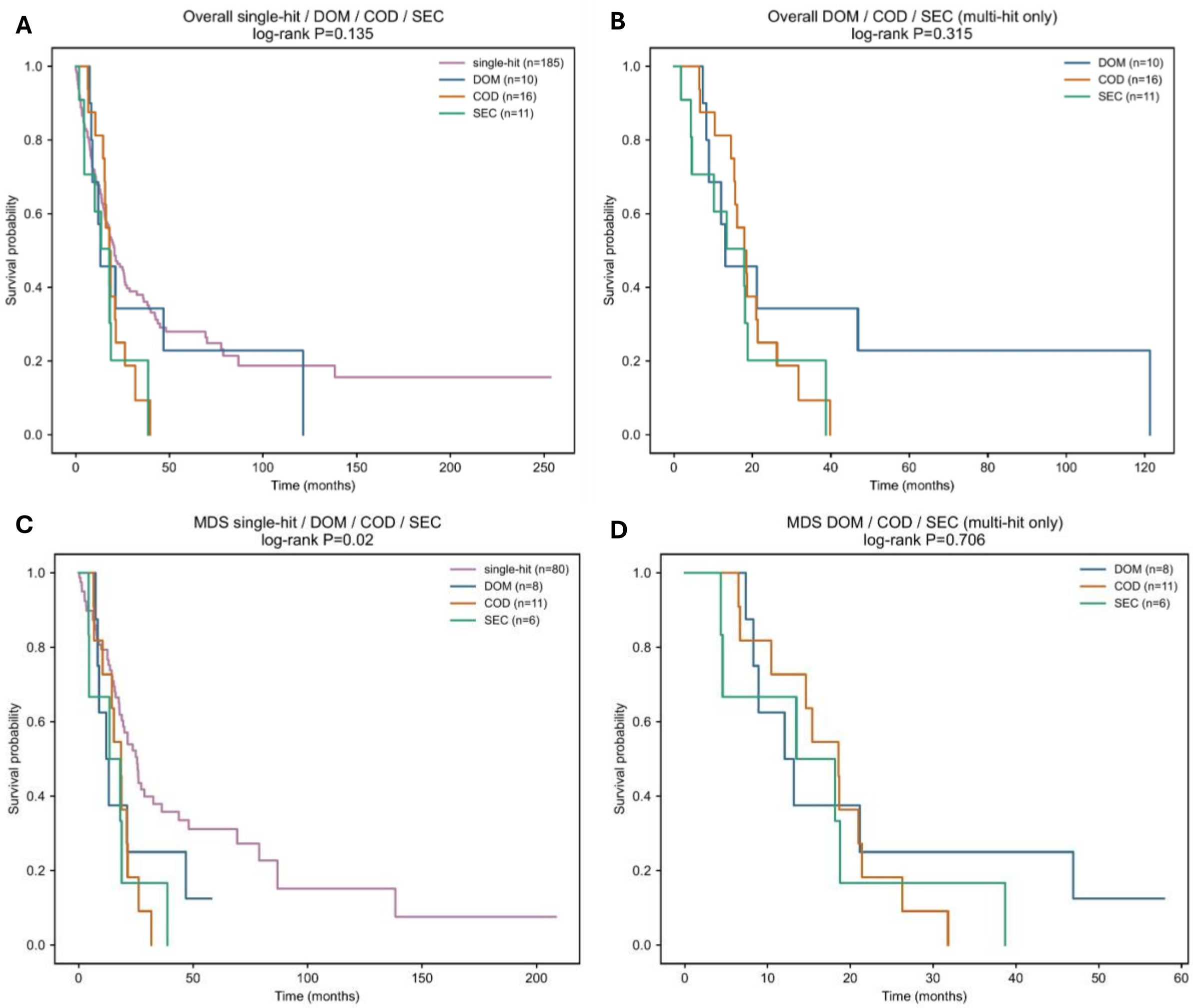

E

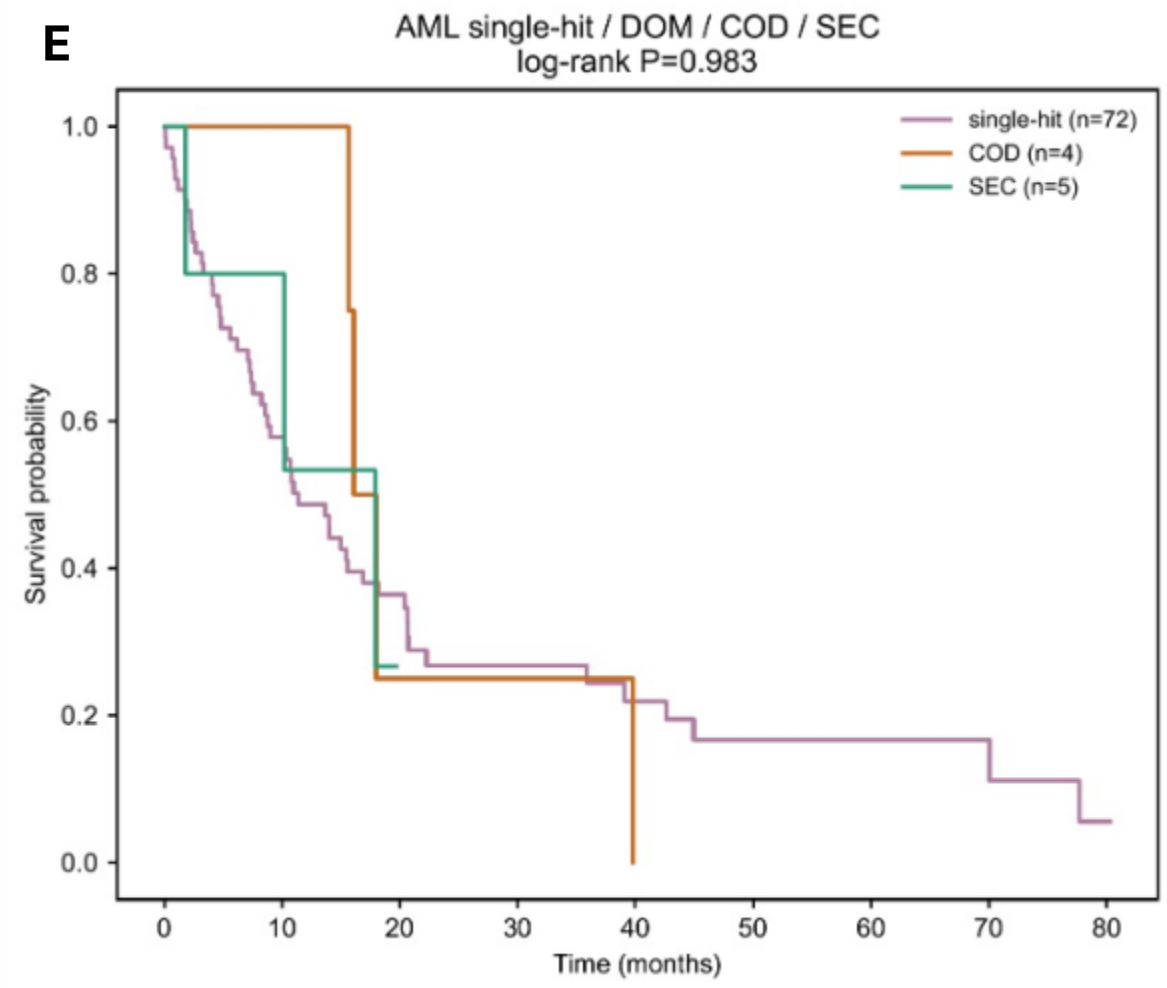
AML single-hit / DOM / COD / SEC
log-rank P=0.983
single-hit (n=72)
COD (n=4)
SEC (n=5)
Survival probability
Time (months)

F

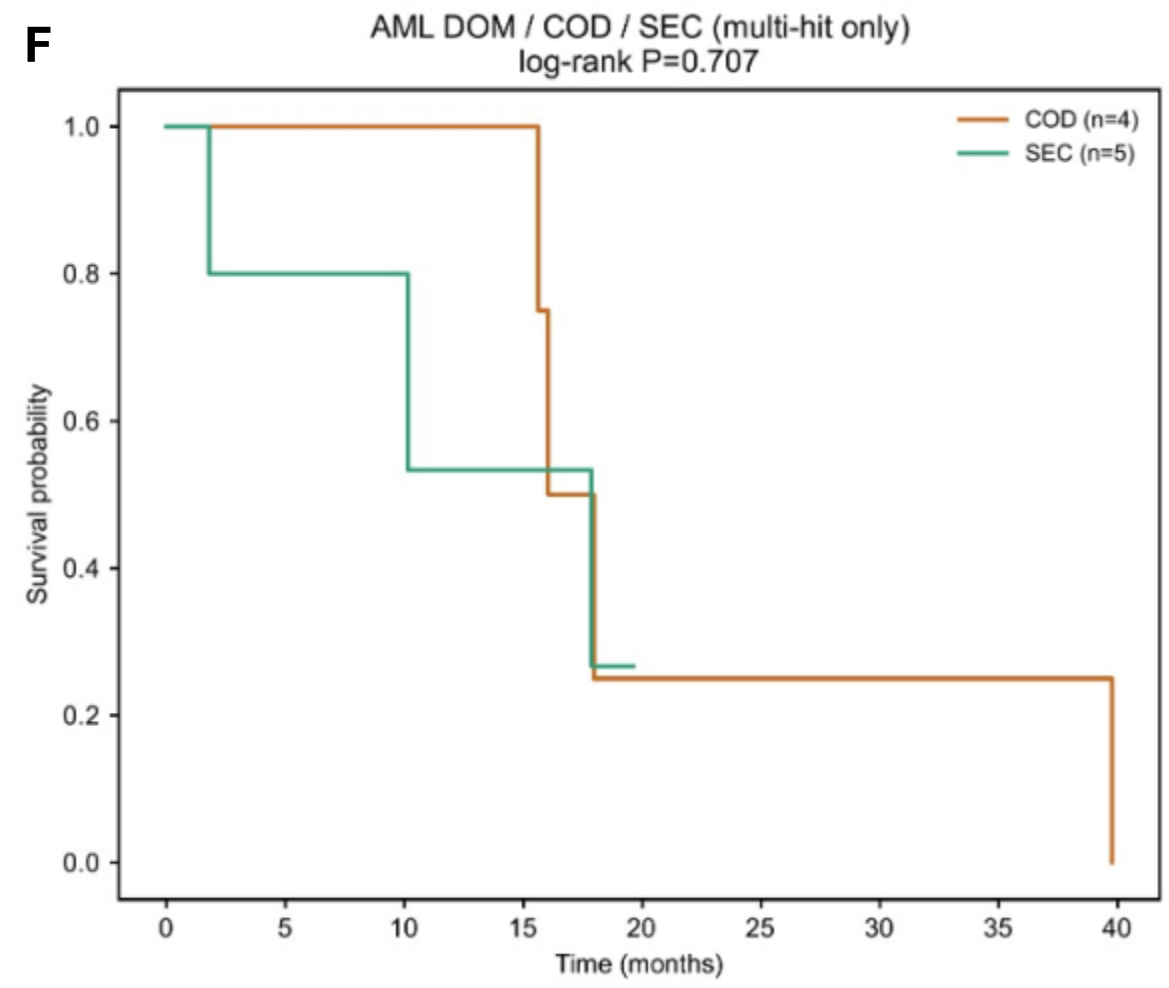
AML DOM / COD / SEC (multi-hit only)
log-rank P=0.707
COD (n=4)
SEC (n=5)
Survival probability
Time (months)
